# Guanfacine Treatment in a Patient with Intravenous Buprenorphine/Naloxone Misuse

**DOI:** 10.1155/2024/6359691

**Published:** 2024-01-19

**Authors:** Sercan Karabulut

**Affiliations:** Centre of Alcohol and Substance Addiction Treatment, Ataturk State Hospital, Antalya, Türkiye

## Abstract

In this case report, we described a patient admitted with buprenorphine/naloxone (BN) misuse, accompanied by intermittent pregabalin misuse, to self-treat the opioid withdrawal symptoms. We treated the withdrawal symptoms after cessation of BN with guanfacine extended-release (XR). To our knowledge, it has been the first case report describing guanfacine-XR in the treatment of BN misuse. Notably, our patient responded to the treatment with a decrease in withdrawal symptoms without any significant side-effect. Although it is not possible to generalize our findings with a single case report, it might be useful to mark guanfacin-XR as a potential treatment agent for opioid use disorders, including patients with synthetic opioid misuse.

## 1. Introduction

Opioid use disorder is one of the major illnesses throughout the world, contributing to negative morbidity, mortality, and social outcomes. Opioid substitution therapy is an effective treatment strategy that reduces illicit opioid use, increases retention rates, decreases criminal activity, and helps to reduce intravenous (IV) opioid use [1–3]. In 2022, The World Drug Report announced that opioids are the second most used drugs, with 61 million opioid abusers globally [4].

Buprenorphine/naloxone (BN) treatment—available in 2/0.5 and 8/2 mg doses—has been used in the treatment of opioid use disorder in Turkey since 2011. This treatment is the only approved opioid replacement therapy option in the country. Buprenorphine is a long-acting partial mu agonist and full kappa antagonist administered sublingually [5]. The naloxone component is intended to block the euphoric/analgesic effects of buprenorphine if the drug is injected [6]. Although the drug's safety profile led the authorities to identify the medication as a first-line option for opioid use disorder, numerous studies reported BN misuse and diversion to the black market [7–9]. Direct analysis of the prevalence of BN misuse is difficult, since the monoproduct (buprenorphine) has been extensively used in some countries. On the other hand, heroin availability may prevent IV drug users to make BN their primary drug, especially in Middle-East regions. Thus, the overall prevalence of BN might be effected by a variety of social, cultural, political, and economical forces [8].

Alpha-2-adrenergic (*α*2A) receptor agonists represent a promising pharmacotherapy for substance use disorders. Given their sympatholytic activity, lofexidine and clonidine were shown to be promising in decreasing symptoms of withdrawal in patients with opioid use disorder. It has been reported that *α*2A receptor agonists reduce noradrenergic hyperactivity observed in opioid withdrawal and are effective on behavior and impulsivity by enhancing regulation in limbic structures as a secondary effect [10]. Guanfacine, on the other hand, is currently Food and Drug Administration-approved for the treatment of attention deficit hyperactivity disorder and hypertension [11]. Given the single-dose advantage, Haney et al. [12] reported in a recent study that guanfacine reduced cannabis withdrawal symptoms (especially irritability and insomnia) and was well tolerated.

In this case, we described a patient admitted with IV BN misuse who appeared to respond to guanfacine XR treatment regarding his withdrawal symptoms. The purpose of this case report is to present data suggesting that guanfacine, an *α*2A receptor agonist, may be an effective treatment option in patients with BN misuse. We hypothesized that the use of guanfacine after buprenorphine cessation would significantly reduce withdrawal symptoms.

## 2. Case Report

In October 2023, a 24-year-old unemployed male presented to the Antalya Ataturk State Hospital outpatient substance addiction treatment clinic for IV BN misuse. He was suffering from pain and restlessness secondary to cessation of BN. In the last 3 months, when he reduced or ceased BN, symptoms, such as restlessness, lacrimation, back and extremity pain, cramps, and insomnia, started to appear within hours. He felt dysphoric and exhausted when he did not use BN. Due to escalating BN use, he has begun to suspect dependency on the medication. The patient had no history of any medical illness in the past.

On the mental status examination, the patient appeared to be tense. Her speech was normal. He was dysphoric. Psychomotor activity was increased, consistent with opiate withdrawal. The content of his thoughts was dominated by an inability to move away from substance use and pessimistic thoughts associated with it. He had no signs of delusion or hallucinations. His attention was prone to be easily distracted.

Throughout his adolescence, there were no symptoms suggestive of borderline personality traits or conduct disorder. His substance use history began at the age of 15 with experimenting with marijuana, introduced by friends out of curiosity. He described feeling euphoria and numbness, using one joint 2–3 times a week. At 18, witnessing someone using heroin in a social setting led him to try it. He had smoked heroin it for a month. Switching to IV use after a friend claimed it provided a better “high,” he continued with 1–2 g of IV heroin daily for the next 5 years (except in his mandatory military service period at 19 for 6 months). He also occasionally used crack cocaine (2 or 3 times a week) in combination with heroin. Infrequently, he encountered and used methamphetamine less than once a month.

About 14 months ago, he was referred to an outpatient substance addiction treatment clinic in another city. An oral BN treatment was initiated at 8 mg daily (2 mg, 4 times a day). After using one or two tablets, he later transitioned to IV use following a friend's suggestion. While he initially described a sense of relaxation and elevation similar to heroin use, recent injections have been solely to alleviate withdrawal symptoms—especially pain. He started with a single IV usage of 2 mg. Over the past 3–4 months, his IV BN use reached 12–16 mg daily with a frequency of four to five injections per day due to tolerance. To mitigate withdrawal symptoms, such as pain, fatigue, restlessness, hot flashes, and insomnia, when using less or abstaining from BN, he started using pregabaline 6 months ago. Subsequently, there was an increase in pregabalin misuse, reaching 3,000 mg daily during this period when he ceased BN use. Within the last month prior to admission, he had not used any pregabaline. Due to conflicts in the marriage and financial difficulties, he separated from his wife. For the past 5 months, he has been living with his parents.

The patient was referred to the inpatient treatment unit. The urine toxicology result was positive for only buprenorphine. The patient's severity of withdrawal symptoms was followed with the Clinical Opiate Withdrawal Scale (COWS) every week. The initial COWS score was 19. He was started on oral BN, which was flexible, with a maximum of 6 mg/day. Patient used 2 mg/day single dose oral BN for 3 days. Afterward, the patient continued to suffer from numbness, pain, psychomotor agitation, and intolerable anxiety, and the treatment was changed with guanfacine, started at 1 mg/day for the first 5 days, and titrated up to 2 mg/day. COWS score was 6 on Day 7. Guanfacine was gradually tapered at the same rate starting from the 10th day and discontinued on Day 20. COWS scores were measured 5 and 3 on Days 14 and 21, respectively. Another drug administered was trazodone (100 mg/day) for insomnia. During the follow-up period, guanfacine was well-tolerated. There was no evidence of fatigue, hypotension, or bradycardia.

Three weeks later, a significant clinical improvement was observed. Withdrawal symptoms gradually decreased. The patient reported a decrease in substance craving. In addition, he was able to take care of himself, read books, and join the group therapy sessions. The patient decided to remain free of medical treatment. As psychiatric symptoms improved, the patient was discharged.

## 3. Discussion

It is worthwhile considering Guanfacine XR as a valuable treatment option to mitigate opioid withdrawal symptoms. The main advantage of this drug is its once-daily usage [11]. Furthermore, in our case, the drug decreased craving severity without serious side effects.

Motivations for using diverted buprenorphine are not well understood. In a sample of more than 1,000 individuals, the main motivation was to self-treat opioid withdrawal symptoms. However, approximately 20% reported using BN to “get high” [13]. The current 4 : 1 BN coformulation ratio seems to be inefficient to reduce the effects of buprenorphine when the combination product is injected. Thus, the BN formulation intends to reduce the risk of misuse at best [8]. In our case, the patient was prescribed BN to be treated for opioid withdrawal symptoms. Afterward, he discovered the fact that the feeling when he misused BN was similar to the feeling of “heroin use.”

Studies with *α*2A receptor agonists showed the efficacy to manage opioid withdrawal symptoms in individuals with opioid use disorder [10, 14]. During acute opioid withdrawal, adrenergic outflow from the locus coeruleus is restored, resulting in a sudden increase in norepinephrine and overstimulation of adrenergic neurons. This leads to various physical manifestations characteristic of opioid withdrawal, especially hypertension, tachycardia, diaphoresis, nausea, and vomiting [14]. A lately published intervention review of *α*2A receptor agonists suggested that the treatment was more effective than placebo in decreasing withdrawal symptoms [15]. Although lofexidine and clonidine were found to be effective, there are some studies comparing clonidine and guanfacine, which found no significant difference in withdrawal severity decrease [16, 17]. Despite its well-known efficacy in opioid withdrawal, promising results have been reported in a recent pilot study on the effectiveness of guanfacine XR in cannabis dependence [18].

This case suggests that Guanfacine XR might be useful in treating individuals with BN misuse. Although the previous study and case reports found concurrent BN misuse in patients with opioid use disorder, the patient that we reported did not have the diagnosis of opioid use disorder, nor did he have concomitant benzodiazepine misuse [19]. In a recent case report showing some similarities to our case, authors reported BN misuse and pregabalin abuse. However, the patient completed the inpatient treatment in our study [20].

A limitation of the case report is the concomitant administration of trazodone. Despite the relatively low dose, other medications generally confound the interpretation of the results. Some might criticize that buprenorphine was used at relatively lower doses (maximum of 6 mg/day) in light of the patient's previous pattern of IV use, which may also complicate interpretation. In addition to these limitations, a more detailed psychometric assessment could have provided additional clinical information about the patient.

Considering the difficulties due to opioid agonist treatment misuse, guanfacine as an opioid detoxification agent seems to be effective to mitigate opioid withdrawal symptoms, including craving. Although it is not possible to generalize observations with a single case, it suggested a potential role on opioid detoxification programs, which might be investigated in further studies. More studies are required to understand the pathophysiology behind BN misuse without significant illicit opioid use. Focusing on studies exploring transdermal buprenorphine use (without the restriction of buprenorphine use) may provide an alternative to misuse.
